# The BrainACT study: acceptance and commitment therapy for depressive and anxiety symptoms following acquired brain injury: study protocol for a randomized controlled trial

**DOI:** 10.1186/s13063-019-3952-9

**Published:** 2019-12-27

**Authors:** Johanne Rauwenhoff, Frenk Peeters, Yvonne Bol, Caroline Van Heugten

**Affiliations:** 1 School for Mental Health and Neuroscience, Department of Psychiatry and Psychology, Maastricht University Medical Centre, P.O. Box 616, 6200 MD Maastricht, The Netherlands; 2 Limburg Brain Injury Centre, P.O. Box 616, 6200 MD Maastricht, The Netherlands; 3 Department of Clinical Psychological Science, Faculty of Psychology and Neuroscience, Maastricht University, P.O. Box 5800, 6202 AZ Maastricht, The Netherlands; 4 Department of Clinical and Medical Psychology, Zuyderland Medical Centre, P.O. Box 5500, 6130 MB Sittard, The Netherlands; 5 Department of Neuropsychology and Psychopharmacology, Faculty of Psychology and Neuroscience, Maastricht University, PO Box 616, 6200 MD Maastricht, The Netherlands

**Keywords:** Acquired brain injury, Acceptance and commitment therapy, Depression, Anxiety, Protocol, RCT

## Abstract

**Background:**

Following an acquired brain injury, individuals frequently experience anxiety and/or depressive symptoms. However, current treatments for these symptoms are not very effective. A promising treatment is acceptance and commitment therapy (ACT), which is a third-wave behavioural therapy. The primary goal of this therapy is not to reduce symptoms, but to improve psychological flexibility and general well-being, which may be accompanied by a reduction in symptom severity. The aim of this study is to investigate the effectiveness of an adapted ACT intervention (BrainACT) in people with acquired brain injury who experience anxiety and/or depressive symptoms.

**Methods:**

The study is a multicenter, randomized, controlled, two-arm parallel trial. In total, 94 patients who survive a stroke or traumatic brain injury will be randomized into an ACT or control (i.e. psycho-education and relaxation) intervention. The primary outcome measures are the Hospital Anxiety and Depression Scale and the Depression Anxiety Stress Scale. Outcomes will be assessed by trained assessors, blinded to treatment condition, pre-treatment, during treatment, post-treatment, and at 7 and 12 months.

**Discussion:**

This study will contribute to the existing knowledge on how to treat psychological distress following acquired brain injury. If effective, BrainACT could be implemented in clinical practice and potentially help a large number of patients with acquired brain injury.

**Trial registration:**

Dutch Trial Register, NL691, NTR 7111. Registered on 26 March 2018. https://www.trialregister.nl/trial/6916.

## Introduction

Patients with acquired brain injury (ABI) have an increased risk of developing emotional disturbances [1]. After surviving a stroke, a quarter of the patients experience depressive symptoms at 6 months post-stroke [2]; almost half of the patients who experienced a traumatic brain injury (TBI) suffer from a major depressive disorder and 38% suffer from anxiety symptoms [3]. Depression has significant effects on the health of patients with ABI. It leads to more hospitalizations, less societal participation, reduces the return-to-work rates, places a greater burden on caregivers, affects social relationships, and has a vast impact on general quality of life [4]. Moreover, anxiety and depressive symptoms have a negative influence on patient rehabilitation [5] and they increase the severity and number of reported health problems such as fatigue, memory, headaches, and concentration problems [6, 7].

Despite their high prevalence and negative consequences with a significant burden of disease, it is still not clear how to best treat post-ABI symptoms of depression and anxiety. Psychopharmacological interventions have mixed results when treating depression in stroke survivors. Additionally, stroke patients seem sensitive to the side effects of psychopharmacological medication [8]. Furthermore, antidepressants have not been shown to be more beneficial than placebo when treating depression following TBI [9]. Cognitive behavioural therapy is widely used for treating depression and anxiety, but there is no strong evidence for its post-stroke effectiveness [10, 11], and research into its effectiveness following TBI is inconclusive [12–15].

A recent type of therapy that has been proven to be effective over a wide range of clinical populations is acceptance and commitment therapy (ACT) [16–19]. ACT is a third-wave cognitive behavioural therapy, which uses commitment and behaviour change strategies to increase psychological flexibility [20]. Psychological flexibility is described as “the ability to contact the present moment more fully as a conscious human being, and to change or persist in behaviour when doing so serves valued ends” [21], p., 7. This results from a combination of the six core processes of ACT: acceptance (accepting positive and negative thoughts to situations one cannot change), defusion (disentanglement of thoughts), sense of self (separating the self from the process of thinking), mindfulness (contact with the present moment), valued living (being aware of what really matters), and committed action (taking action guided by one’s values) [21]. Although symptom reduction is not the primary treatment goal, an increase in psychological flexibility is often accompanied by a decrease in symptomatology [22, 23]. ACT is an interesting approach for people with ABI who suffer from psychological distress [24]. Instead of applying cognitive restructuring techniques as used in traditional cognitive therapy, ACT focusses on learning to accept both the negative and positive thoughts and feelings related to those circumstances that cannot be changed or controlled [25]. It enables the individual to carry out value-based behaviour while feeling distressed [25]. Higher levels of acceptance and valued living have been associated with better psychological outcomes in patients with ABI [26–28].

Currently, there is some evidence that people who developed depressive or anxiety symptoms after ABI can benefit from ACT. Majumdar and Morris [29] performed a randomized controlled trial (RCT) in which an ACT group-intervention of four weekly didactic PowerPoint sessions was compared to treatment as usual in stroke survivors. They found that people in the ACT intervention showed a significant reduction in depression and increased hopefulness and self-rated health. However, there were no differences identified in relation to anxiety or quality of life. Graham, Gillanders, Stuart, and Gouick [30] described a case study in which a stroke patient no longer experienced chest pain, had a decrease in anxiety and depressive symptoms, and improved illness perception and psychological flexibility following an ACT intervention. Whiting, Deane, McLeod, Ciarrochi and Simpson [31] performed a pilot RCT comparing an ACT group intervention with befriending therapy in patients with severe TBI. Their results show that ACT decreased the levels of anxiety and depression significantly compared to the befriending treatment. However, it could not be confirmed that the mechanism through which this decrease was elicited was psychological flexibility [31]. These findings should, however, be considered in light of the small sample size of the study and the restricted sample characteristics.

These studies provide preliminary support for the hypothesis that ACT can be effective in treating psychological distress following ABI. With the current BrainACT study, we aim to investigate whether an ACT intervention, adapted for the needs and possible cognitive deficits of people with ABI, results in decreased anxiety and depressive symptoms compared to a control intervention. Furthermore, we will examine ACT-related processes, quality of life, social participation, and the cost-effectiveness of both interventions.

Research questions addressed in the study are:

1. Does ACT lead to a greater reduction of depressive and anxiety symptoms in patients with ABI compared to an active control intervention (i.e. psycho-education and relaxation training)?
2. Is ACT more cost-effective in comparison to an active control intervention?
3. Is the (potential) reduction of anxiety and depressive symptoms mediated by an increase in psychological flexibility, acceptance, valued living, and/or cognitive defusion in the ACT group?
4. Does ACT lead to higher levels of participation and quality of life, compared to an active control condition?

## Methods

### Study design

**Fig. 1:**
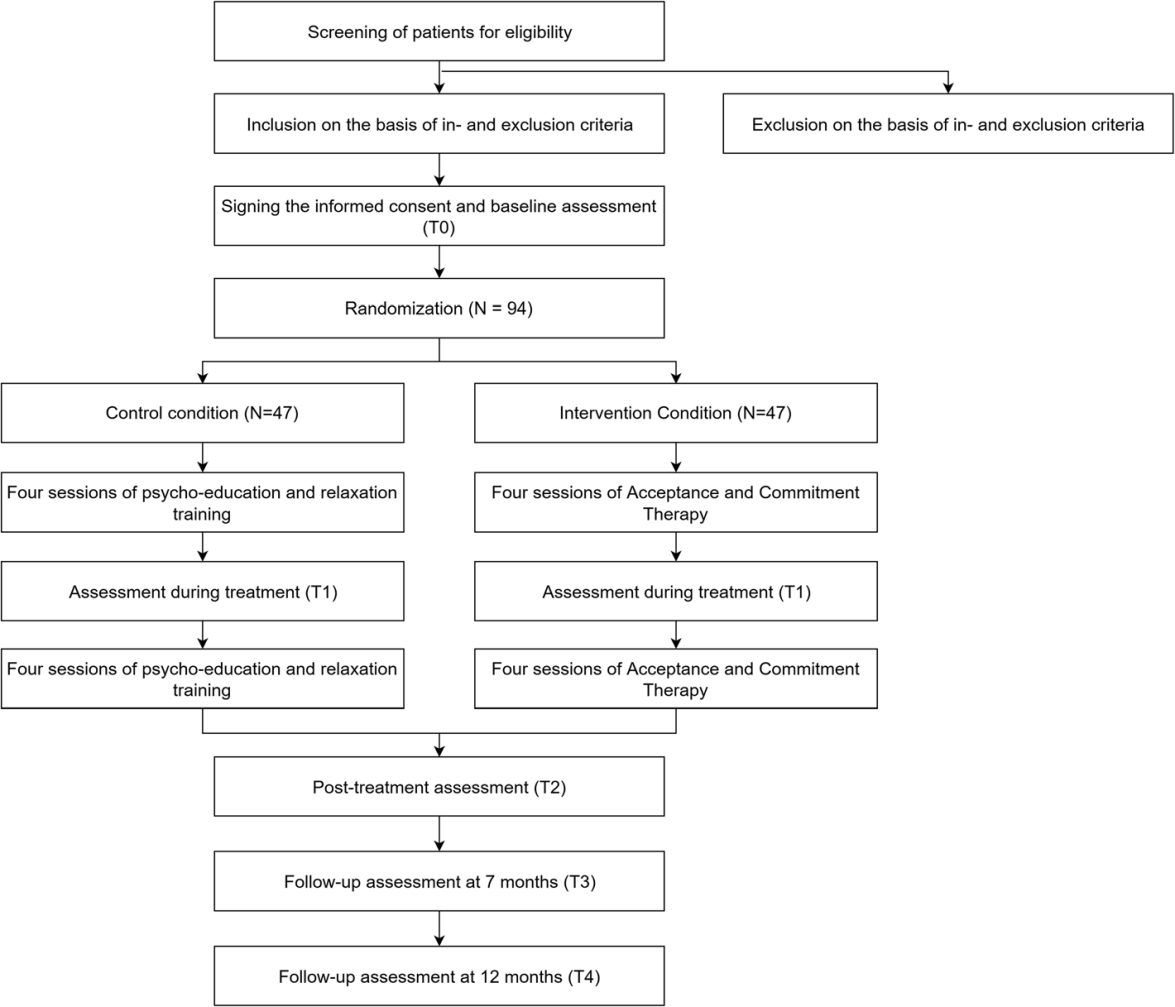
Design of the study: multicenter, randomized, controlled, two-arm parallel trial

The design of the study is a multicenter, randomized, controlled, two-arm parallel trial in which an 8-week ACT intervention will be compared to an active control intervention, namely psycho-education combined with relaxation training. Outcome measures will be collected at baseline (T0), after 1 month (T1; during treatment), and after 3 months (T2; post-treatment). There will be follow-up measurements at 7 months (T3) and 12 months (T4). See Fig. 1 for the flow chart of the study. Ethics approval for the study has been given by the medical research ethics committee of Maastricht University Medical Centre and Maastricht University and the local committees of participating clinical centres. The study is registered in the Dutch Trial Register (TC: 7111).

### Participants

A total of 94 survivors of TBI or stroke will be recruited from rehabilitation or medical psychology departments at hospitals in the Netherlands. Patients who have been referred to a psychologist will be considered for participation. Possible participants will be screened and informed about the study by their psychologists for eligibility. Inclusion criteria are (1) having sustained any type of stroke or TBI, which is objectified by a neurologist; (2) scoring > 7 on the depression and/or anxiety subscale of the Hospital Anxiety and Depression Scale (HADS); (3) being 18 years or older; (4) having a stable use of psychotropic medication (such as antidepressants) for the duration of the study and stable use of antidepressants during 4 weeks prior to the start of the study; (5) being able to access the Internet and a computer because treatment materials such as patient videos are shown via the Internet; (6) mastering the Dutch language sufficiently to benefit from treatment; and (7) giving informed consent. Exclusion criteria are (1) history of brain injury or any neurological disorder other than a stroke and TBI; (2) pre-morbid disability as assessed by the Barthel Index (score < 19/20); (3) severe co-morbidity, for which treatment is given at the moment of inclusion, that might affect the outcome; (4) presence of a DSM 5-based mood and/or anxiety disorder for which pharmacological and/or psychological treatment was necessary during the onset of the brain injury; and (5) attendance in a previous ACT intervention for comparable problems in the year proceeding entry to the current study.

### Randomization and blinding

Participants who are eligible and agree to participate in the study are randomly assigned to either the intervention group or the control group with an allocation ratio of 1:1. An independent third person will randomize participants centrally using computerized block randomization. The block size is six and the randomization scheme includes pre-stratification on type of brain injury (TBI versus stroke). The randomization scheme has been developed with the use of a random generator (www.random.org). After randomization, an opaque, sealed envelope will be prepared containing information about the group allocation of the participant by the independent third person. After the first measurement time point (T0) the researcher will open the sealed envelope and reveal the treatment allocation to the patient and psychologist.

The trained research assistants that administer the questionnaires will be blinded to treatment allocation (i.e. intervention or control) and should never be unblinded. Participants will be asked not to tell research assistants about the treatment they received. In order to check the success of blinding, at T4 the research assistants will be asked to indicate group allocation for all participants who completed the trial. The research assistants will choose one of the following options: “Intervention group”, “Control group”, or “I don’t know”. Participants will not be blinded to treatment allocation. Data will be analysed by the principal investigator (JR) who will be blinded to treatment allocation while performing the analyses.

### Primary outcome measures

#### Psychological distress

The subscales of the HADS will be used to measure anxiety and depressive symptoms over the past 7 days. The scores for the scales, seven items each, range from 0 to 21 with higher scores indicating higher levels of depression or anxiety. This questionnaire has been validated in a sample of patients with TBI [32]. Furthermore, there was good internal consistency for both subscales (Cronbach α depression scale, 0.81; anxiety scale, 0.84) in patients with stroke [33]. The Depression Anxiety Stress Scales-21 (DASS-21) will be used to measure the participants’ levels of anxiety, depression, and stress. It consists of 21 items, which are rated on a 4-point Likert scale. The score ranges from 0 to 63 with higher scores indicating greater levels of depression, anxiety, or stress. The questionnaire has been validated in a sample of patients with TBI. The internal consistency was good for the three scales (Cronbach α depression scale, 0.90; stress scale, 0.89; anxiety scale. 0.82) [34]. The primary outcome is the difference between groups in change in mood and anxiety symptoms over all time points, as measured by the total scores of the respective subscales of the HADS and DASS-21.

#### Cost effectiveness

Two questionnaires will be used to measure cost effectiveness: the 5-level Euroqol-5 dimensions (EQ-5D-5 L) and a 16-item cost questionnaire. The EQ-5D-5 L consists of five questions measuring health status. The dimensions covered are mobility, self-care, daily activities (such as work, housework, study, and leisure activities), pain or discomfort, and anxiety or depression. These domains are rated as “no problem”, “slight problem”, “‘moderate problem”, “severe problem”, or “unable to do”. Consequently, the EQ-5D-5 L can distinguish between different health states. For each of the different states a weight is contributed based on the valuation given by the general population [35]. The healthcare costs of participants will be measured with a self-report cost questionnaire, which is constructed to collect cost data from a societal perspective, based on the steps described by Thorn et al. [36]. The cost questionnaire used in this study is based on a questionnaire used in the study of Kootker et al. [11]. Several questions are altered based on response patterns and analyses in this prior study. The primary cost-effectiveness outcome is the change in the amount of care used between groups over all time points, as measured by the total scores of the cost-questionnaire and the EQ-5D-5 L.

### Secondary outcome measures

#### Participation

The Utrecht Scale for Evaluation of Rehabilitation-Participation (USER-P) will be used to measure three aspects of participation: frequency of behaviours, participation restrictions experienced due to health condition, and satisfaction with participation. The frequency scale measures the objective level of participation, while the restrictions and satisfaction scales offer an insight into the subjective rating of participation. The questionnaire consists of 31 items. A score ranging from 0 to 100 is calculated for each scale. Higher scores indicate more participation, less restriction, and more satisfaction. It is a valid and reliable measure for patients with ABI; the internal consistency is good (Cronbach α = 0.70–0.91) [37].

#### Health status

The Short Form Survey (SF-12) will be used to measure the health status of the participants. The total score ranges from 0 to 100 and a higher score indicates better health status. The SF-12 has been validated for use in TBI research [38]. The secondary outcome is the difference between groups in change in participation and health status over all time points, as measured by the total scores of the respective subscales of the USER-P and SF-12.

### Secondary process measures

#### Psychological flexibility

The Acceptance and Action Questionnaire II (AAQ-II) is a seven-item questionnaire measuring psychological flexibility. The items are scored on a 7-point Likert scale and the total score ranges from 0 to 49 with a higher score indicating more psychological flexibility. The internal consistency of the AAQ-II is good (Cronbach α = 0.84) [39]. Furthermore, the questionnaire has been validated in a sample of patients with ABI [40]. The Acceptance and Action Questionnaire after brain injury (AAQ-ABI) measures psychological flexibility about the thoughts and feelings related to the brain injury, while the AAQ-II measures psychological flexibility around general psychological distress. The AAQ-ABI consists of seven items, which are scored on a 5-point Likert scale. The score ranges from 0 to 36 with higher scores indicating greater psychological flexibility. The questionnaire has good internal consistency (Cronbach α = 0.89) [40].

#### Valued living

The Valued Living Questionnaire (VLQ) is a two-part instrument that measures valued living. First, the participant rates the importance of ten value domains on a 10-point Likert scale. Second, participants rate how consistently he or she has lived in accordance with their values within these domains. Scores from both parts are used to calculate a valued living component. The internal consistency of the valued living component has been demonstrated as adequate (Cronbach α = 0.74) [41]. The VLQ has been used in earlier research to measure valued living in patients with TBI [27].

#### Cognitive fusion

The Cognitive Fusion Questionnaire (CFQ-7) measures cognitive fusion on a 7-point Likert scale. Scores range from 0 to 49. The higher the score, the more fused one is with one’s thoughts. Earlier research has shown that the CFQ has good factor structure, validity, reliability, and stability over time [42]. The questionnaire has been validated in healthy individuals and in patients [42, 43]. The secondary process outcome is the difference between groups in change in psychological flexibility, valued living, and cognitive fusion over all time points, as measured by the total scores of the respective subscales of the AAQ-II, AAQ-ABI, VLQ, and CFQ-7.

### Personal and brain-injury-related characteristics and feasibility

Participants will fill out a demographic questionnaire at the start of the study. This questionnaire will register age, gender, employment, and education. Injury-related factors will be obtained from the medical files of the participants. These include type of brain injury, time since brain injury, severity of injury, and affected hemisphere of the brain. After ending the treatment, the participant’s opinion of and satisfaction with the ACT (treatment feasibility) will be questioned by means of a short semi-structured interview.

### Procedure

**Fig. 2:**
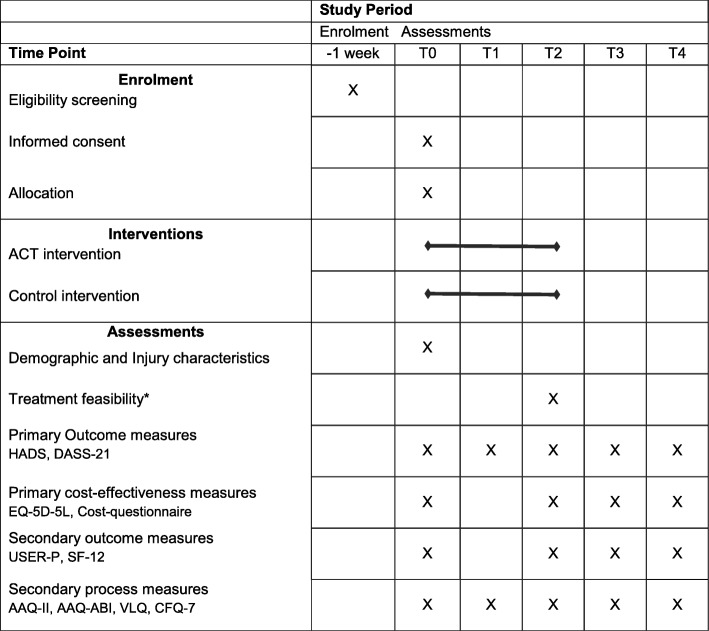
Standard protocol items: recommendation for interventional trials (SPIRIT) diagram

Participants will be recruited at participating hospitals and rehabilitation centers in the Netherlands. To prevent unnecessary burden, the psychologists will check the inclusion and exclusion criteria. If the patient is willing to participate the investigator will meet with the patient (at the patient’s home, the hospital, or the university, as preferred by the patient) to discuss the participation in the study and answer questions. If all is clear the informed consent form will be signed by the patient and the researcher (see Additional file 2). Subsequently, the first measurements will take place (T0) and the participant will be randomly allocated to the experimental group receiving ACT or to the active control group receiving psycho-education. The therapy sessions will be scheduled with the psychologist or healthcare professional. One month after the start of the intervention both groups will fill in the questionnaires for T1 (see Fig. 2). Questionnaires will be filled in on paper and measurements will be conducted by a research assistant blinded to allocation. After 3 months, when the treatment program is completed, the participants will fill in the questionnaires for T2. There will be follow-up measurements at 7 months (T3) and 12 months (T4). Again, these measurements will be conducted by a research assistant blinded to allocation. Research assistants will enter the data and data entry will be periodically checked during the monitoring visits. Follow-up appointments will be planned at the end of each visit. The research assistant will remind participants of their appointment by telephone or e-mail 1 week before the appointment is due. During the assessments, the researcher and research assistants will be alert for any adverse events such as suicidality. All adverse events related to mood complaints reported spontaneously by the subject or observed will be systematically collected and reported to the medical research ethics committee. Based upon these events the decision may be made to discontinue participation in the study. Furthermore, participants can leave the study without any consequences, at any time, for any reason if they wish to do so. The investigator can decide to withdraw a subject from the study for urgent medical reasons.

### Interventions

#### BrainACT intervention

The ACT intervention involves eight weekly individual sessions of 60 min during a period of 3.5 months. The first four sessions are weekly, thereafter the sessions will be biweekly, with a 3-week break between the seventh session and last session. Participants will do homework exercises of around 30 min for 6 days a week. Homework consists of reading or listening to summaries of the sessions, practising skills, and doing mindfulness exercises. At the start of the intervention, participants will receive a workbook with instructions that they can read at home after each session. We use an eight-session protocol (see Table 1) based on Jansen and Batink [44], Luoma, Hayes, and Walser [45], and Whiting et al. [31] in which all six ACT core processes are addressed. Psychologists are free to decide on the order of the sessions. We altered the treatment for people with ABI as suggested by Kangas and McDonald [24] and Broomfield et al. [46]. An expert group of psychologists experienced in ACT and/or in working with people with ABI gave further advice on the alterations. The possible cognitive deficits of the participants are taken into account and brain-injury-related topics are discussed during the treatment. A treatment protocol is specified to ensure comparability of the ACT intervention across settings. The intervention will be provided individually by certified psychologists who completed an ACT training course of at least 5 days and are experienced in working with patients with ABI. The therapy will take place at hospital outpatient facilities.

**Table 1:** Overview of the BrainACT treatment programme

Topic	Content
Values	Value exploration and difference between goals and actions
Committed action and Mindfulness	Committed action on the long and short term in relation to values, education about mindfulness, and practising contact with the present moment
Effect of control	Creative hopelessness; the undeniability of human suffering and the long-term consequences of trying to control it
Acceptance	Introducing acceptance as an alternative to control
Defusion	Changing the relationship with thoughts, naming the mind
Self-as-context	Changing the relationship with thoughts about oneself and introducing the constant self
Defusion and Mindfulness	Review and exercises on defusion and mindfulness
Psychological flexibility	Review of the different core components, explanation on how these skills together lead to psychological flexibility, and preparation on relapse and setbacks

#### Active control intervention

The control condition (see Table 2 for an overview) consists of an eight-session psycho-education intervention combined with relaxation training with a duration of 1 h. A Cochrane review concluded that active information provision is able to reduce depression and anxiety after brain injury [47]. Furthermore, earlier research has shown that relaxation exercises reduce post-stroke anxiety [48]. The psycho-education is based on the existing module *Niet rennen maar plannen* (don’t run, plan) [49], which is a training and education programme focussing on cognitive rehabilitation for patients with brain damage and mild cognitive problems. The intervention aims to offer information and education about brain injury and its consequences. Relaxation exercises consist of progressive muscle relaxation [50] and autogenic training [51]. Participants will do homework exercises of around 30 min for 6 days a week. A treatment protocol is specified to ensure comparability of the education intervention across settings. This intervention will be provided individually by a healthcare professional with experience in working with patients with brain injury (e.g. psychologist, social worker, occupational therapist, psychological assistant, but not the psychologist providing the ACT intervention) at hospital outpatient facilities.

**Table 2:** Overview of the psycho-education and relaxation treatment program

Session number	Content
1	Psycho-education on the basic functions of the brain and causes of brain injury
2	Psycho-education on relaxation training and a progressive muscle relaxation exercise
3	Psycho-education on the consequences of brain injury and a progressive muscle relaxation exercise
4	Psycho-education on changes in behaviour and mood following brain injury and an autogenic training exercise
5	Psycho-education on fatigue following brain injury and an autogenic training exercise
6	Psycho-education on memory problems following brain injury. Participants can choose between autogenic training or a progressive muscle relaxation exercise. This technique will also be used in the following sessions
7	Psycho-education on information processing and planning and an autogenic training exercise or a progressive muscle relaxation exercise
8	Review of the different sessions, wrap up, and an autogenic training exercise or a progressive muscle relaxation exercise

#### Adherence

To ensure adherence to the intervention protocol, all therapists (for the intervention and control condition) will be trained by the principal investigator (JR). Furthermore, to examine the adherence during the different sessions, therapists have to fill in a checklist after every session. They tick off which exercises were done according to the treatment protocol and which exercises were added or left out.

### Sample size calculation

The sample size calculation was performed using G*Power based on a repeated measures design. With a large effect size of 0.4, alpha value of 0.05, power value of 0.80, two groups (intervention and control), and five measurements, at least 80 participants are required. With an expected drop-out rate of 15%, 94 participants need to be recruited.

### Statistical analysis

The statistical analysis can be divided into four parts. First, the baseline characteristics will be described. Second, between-group differences at baseline will be analysed using the chi-square test and independent samples *t* test to verify the success of randomization. Furthermore, groups will be compared on primary outcome measures at T2 using the chi-square test and independent sample *t* test. Linear mixed models for repeated measures will be used to study the differences between groups with the primary and secondary outcome measures as dependent variables. Models will include main effects of the intervention (ACT or psycho-education) and time (T0, T1, T2, T3, and T4) as categorical variables and the interaction effect of intervention and time. Baseline-adjusted mean differences between groups at each time point with 95% confidence intervals will be calculated. The effects of the intervention will be analysed according to the intention-to-treat principle. Alpha will be set at 0.05. All quantitative analyses will be conducted using SPSS software version 25 [52]. Clinically significant change will be determined on the primary outcome measures [53] to gain insight into clinical improvement on an individual level. The first step is to identify patients who “recovered” by using the cut-off scores of the primary outcome measures (HADS and DASS-21). The second step is to calculate the reliable change index, which identifies the patients with a significant improvement. Patients who both recovered and showed a significant improvement will be considered clinically significantly improved. The number of clinically significantly improved patients will be compared between interventions. The trial-based economic evaluation will involve a combination of cost-effectiveness analysis and cost-utility analysis.

### Ethical considerations and dissemination

Data will be handled confidentially and reporting will be coded. Each participant is given a personal code, which can only be linked to a person when the coding key is known. Collected data and personal information will be stored separately. The coded dataset will be available to the research team, healthcare inspectorate, study monitors, and members of the medical research ethics committee. The handling of personal data will comply with the General Data Protection Rule and the Research Data Management Code of Conduct of Maastricht University. Data will be used for future studies if the patient agreed upon this in the informed consent form. Data and material will be stored for 15 years in a repository of Maastricht University and can be made available to researchers upon request.

Participants will be compensated for any harm or injury due to participation in the study. The sponsor/investigator has liability insurance, which is in accordance with article 7 of the Medical Research involving Human Subjects Act. All adverse events will be followed until they have abated, or until a stable situation has been reached. Depending on the event, follow up may require additional tests or medical procedures as indicated, and/or referral to the general physician or a medical specialist. Any further treatment required for mood complaints after termination of the intervention will be arranged within the healthcare system. Adverse events will not be reported in the publication unless an adverse event occurs often and will, therefore, lead to a bias.

Ethics approval for the study has been given by the medical research ethics committee of Maastricht University Medical Centre and Maastricht University (committee reference number NL65349.068.18). Any protocol modifications will be communicated to the ethics committee. The study will be monitored by the Clinical Trial Centre Maastricht. In every site, there will be a site initiation visit, two monitoring visits, and a close-out visit. The monitoring will be performed independently of investigators and the sponsor.

In accordance with the Central Committee on Research Involving Human Subjects statement on publication policy, the researchers aim to publish the results of the study (positive or negative) in international, peer-reviewed journals. Furthermore, results will be presented at professional conferences and provided to study participants upon request. The standard protocol items: recommendation for interventional trials (SPIRIT) checklist for this trial is shown in Additional file 1.

## Discussion

Currently, there are no evidence-based treatment options for people with ABI who experience psychological distress. To our knowledge, no large RCTs have been conducted investigating the effectiveness of ACT for anxiety and depressive symptoms following TBI or stroke. However, earlier studies have shown promising results [29–31]. The aim of the proposed study is to investigate the clinical and cost effectiveness of an upcoming cognitive behavioural therapy, ACT, for anxiety and depressive symptoms following ABI.

We have taken into account recommendations made by Ost [18], to enhance the quality of studies in the field of ACT, such as having an active control intervention, timing of follow-up measurements, and choice of outcome measures. As described earlier, the aim of ACT is to change the functions and context of behaviour and thoughts; symptom reduction is not a treatment outcome [21]. However, the primary outcome measures in this study are the HADS and DASS-21 (measures of anxiety and depression). We have chosen these outcome measures for several reasons. First, a reduction in symptomatology (such as a decrease in psychological distress) is still regarded as a beneficial treatment outcome in ACT next to an increase in psychological flexibility. Second, when psychological flexibility increases, a decrease is often observed in psychological distress [22, 23]. We expect that a decrease in anxiety and depression is elicited through an increase in psychological flexibility. Psychological flexibility is measured with the secondary process outcomes. Last, choosing the HADS and DASS-21 as primary outcome measures will aid comparability with studies outside the field of ACT.

This study has several strengths. First, it has a relatively large sample size. Second, the study has a long follow-up period. Third, the ACT intervention will be compared to an active control intervention that has been shown to have positive effects on anxiety and depressive symptoms following brain injury. Fourth, the ACT treatment protocol was adapted to the needs and possible cognitive deficits of patients with brain injury. These adaptations were based on suggestions made in previous literature and knowledge gained in clinical practice. Fifth, the effectiveness of the ACT intervention will be investigated in a quantitative as well as qualitative manner.

This study also has certain limitations. First, its clinical nature prevents blinding of the participants and the therapists. However, the assessments will be performed by research assistants unaware of group allocation. Second, participant recruitment and loss to follow up are known to be problematic in studies with patients with ABI. To aid study adherence research assistants will be trained and the outcome assessments will take place at the participants’ home, the University, or the hospital, as preferred by the participant.

The results of this study notwithstanding should contribute to the limited knowledge on how to treat psychological distress following ABI. If effective, the BrainACT intervention can be implemented in clinical practice. Given the high prevalence of anxiety and depressive symptoms, it has the potential to help a large number of patients with ABI.

### Trial status

Recruitment started on 24 April 2019 and is expected to be completed by 1 May 2021. Protocol version 8.0, date 21 October 2019.

## Supplementary information

**Additional file 1.** SPIRIT 2013 checklist.

**Additional file 2. ***Toestemmingsformulier proefpersoon*.

## Data Availability

Not applicable.

## Abbreviations

AAQ-ABI: Acceptance and Action Questionnaire after brain injury
AAQ-II: Acceptance and Action Questionnaire II
ABI: Acquired brain injury
ACT: Acceptance and commitment therapy
CFQ-7: Cognitive Fusion Questionnaire
DASS-21: Depression Anxiety Stress Scale
EQ-5D-5 L: Five-dimensional five-level EuroQol
HADS: Hospital Anxiety and Depression Scale
RCT: Randomized controlled trial
SF-12: Short Form Survey
TBI: Traumatic brain injury
USER-P: Utrecht Scale for Evaluation of Rehabilitation-Participation
VLQ: Valued Living Questionnaire
