# Supplementary material for: The BrainACT study: acceptance and commitment therapy for depressive and anxiety symptoms following acquired brain injury: study protocol for a randomized controlled trial
Source: Trials. 2019 Dec 27;20:773. doi: 10.1186/s13063-019-3952-9 (PMC6935100; doi:10.1186/s13063-019-3952-9)
Supplement: Supplementary file 2 — Additional file 2. Toestemmingsformulier proefpersoon. [file 13063_2019_3952_MOESM2_ESM.docx]

**Toestemmingsformulier proefpersoon**

De behandeling van angst en somberheid na hersenletsel

- Ik heb de informatiebrief gelezen. Ook kon ik vragen stellen. Mijn vragen zijn voldoende beantwoord. Ik had genoeg tijd om te beslissen of ik meedoe.
- Ik weet dat meedoen vrijwillig is. Ook weet ik dat ik op ieder moment kan beslissen om toch niet mee te doen of te stoppen met het onderzoek. Daarvoor hoef ik geen reden te geven.
- Ik geef toestemming voor het opvragen van informatie uit mijn medisch dossier over de aard van mijn hersenletsel.
- Ik weet dat voor de controle van het onderzoek sommige mensen toegang tot al mijn gegevens kunnen krijgen. Die mensen staan vermeld in deze informatiebrief. Ik geef toestemming voor die inzage door deze personen.
- Ik geef toestemming voor het verzamelen en gebruiken van mijn gegevens voor de beantwoording van de onderzoeksvraag in dit onderzoek.
- Ik geef □ **wel**

□ **geen** toestemming om mij na dit onderzoek opnieuw te benaderen voor een vervolgonderzoek.

- Ik geef □ **wel**

□ **geen** toestemming om mijn persoonsgegevens langer te bewaren en te gebruiken voor toekomstig onderzoek op het gebied van behandelingen voor patiënten met hersenletsel.

- Ik wil meedoen aan dit onderzoek.

Naam proefpersoon:

Handtekening: Datum : __ / __ / __

-----------------------------------------------------------------------------------------------------------------

Ik verklaar dat ik deze proefpersoon volledig heb geïnformeerd over het genoemde onderzoek.

Als er tijdens het onderzoek informatie bekend wordt die de toestemming van de proefpersoon zou kunnen beïnvloeden, dan breng ik hem/haar daarvan tijdig op de hoogte.

Naam onderzoeker (of diens vertegenwoordiger):

Handtekening: Datum: __ / __ / __

-----------------------------------------------------------------------------------------------------------------

Aanvullende informatie is gegeven door:

Naam:

Functie:

Handtekening: Datum: __ / __ / __

-----------------------------------------------------------------------------------------------------------------
